# The Development, Validation, and User Evaluation of Foodbook24: A Web-Based Dietary Assessment Tool Developed for the Irish Adult Population

**DOI:** 10.2196/jmir.6407

**Published:** 2017-05-11

**Authors:** Claire M Timon, Richard J Blain, Breige McNulty, Laura Kehoe, Katie Evans, Janette Walton, Albert Flynn, Eileen R Gibney

**Affiliations:** ^1^ UCD Institute of Food and Health University College Dublin Dublin Ireland; ^2^ UCC School of Food and Nutritional Sciences University College Cork Cork Ireland

**Keywords:** diet records, Internet, validity, biomarkers, method acceptability, adults

## Abstract

**Background:**

The application of technology in the area of dietary assessment has resulted in the development of an array of tools, which are often specifically designed for a particular country or region.

**Objective:**

The aim of this study was to describe the development, validation, and user evaluation of a Web-based dietary assessment tool “Foodbook24.”

**Methods:**

Foodbook24 is a Web-based, dietary assessment tool consisting of a 24-hour dietary recall (24HDR) and food frequency questionnaire (FFQ) alongside supplementary questionnaires. Validity of the 24HDR component was assessed by 40 participants, who completed 3 nonconsecutive, self-administered 24HDR using Foodbook24 and a 4-day semi-weighed food diary at separate time points. Participants also provided fasted blood samples and 24-hour urine collections for the identification of biomarkers of nutrient and food group intake during each recording period. Statistical analyses on the nutrient and food group intake data derived from each method were performed in SPSS version 20.0 (SPSS Inc). Mean nutrient intakes (and standard deviations) recorded using each method of dietary assessment were calculated. Spearman and Pearson correlations, Wilcoxon Signed Rank and Paired *t* test were used to investigate the agreement and differences between the nutritional output from Foodbook24 (test method) and the 4-day semi-weighed food diary (reference method). Urinary and plasma biomarkers of nutrient intake were used as an objective validation of Foodbook24. To investigate the user acceptability of Foodbook24, participants from different studies involved with Foodbook24 were asked to complete an evaluation questionnaire.

**Results:**

For nutrient intake, correlations between the dietary assessment methods were acceptable to very good in strength and statistically significant (range *r*=.32 to .75). There were some significant differences between reported mean intakes of micronutrients recorded by both methods; however, with the exception of protein (*P*=.03), there were no significant differences in the reporting of energy or macronutrient intake. Of the 19 food groups investigated in this analysis, there were significant differences between 6 food groups reported by both methods. Spearman correlations for biomarkers of nutrient and food group intake and reported intake were similar for both methods. A total of 118 participants evaluated the acceptability of Foodbook24. The tool was well-received and the majority, 67.8% (80/118), opted for Foodbook24 as the preferred method for future dietary intake assessment when compared against a traditional interviewer led recall and semi-weighed food diary.

**Conclusions:**

The results of this study demonstrate the validity and user acceptability of Foodbook24. The results also highlight the potential of Foodbook24, a Web-based dietary assessment method, and present a viable alternative to nutritional surveillance in Ireland.

## Introduction

### Background

Dietary assessment methodologies are known to have both strengths and limitations [1]. Some of the methodological caveats among current dietary assessment methods include the participant burden, reliance on participant’s honesty and ability to remember food and drinks consumed, and their individual portion sizes [2,3]. Cost, particularly when large-scale epidemiological studies and national nutrition surveys are concerned, can be another limiting factor [1]. The 24-hour dietary recall (24HDR) method is associated with low participant burden and can provide reliable intake data with minimal bias [3]. However, recalls can be expensive, time consuming to administer, and require skilled nutritionists or dietitians [4].

The application of technology in dietary assessment has made it possible to minimize the reliance on trained interviewers and instead facilitate automated self-administered 24HDR via Web-based platforms and mobile phone apps [5]. Web-based methodologies facilitate the collection of dietary intake across many geographic locations [6], from large cohorts [7], and are often preferred by participants compared with the traditional methods [8,9]. An example of a successful Web-based 24-hour recall tool is the ASA24 developed by the National Cancer Institute, USA. From its launch in 2009, more than 200 researchers have used ASA24 to carry out over 120,000 recalls [4].

### Biomarker Analysis

A prerequisite for the acceptance and use of such Web-based dietary assessment tools is their validity. It is vital that new tools and methods measure what they are designed to measure. Assessing the relative validity of a new method or tool can be achieved by comparing intakes recorded by a new method to intakes derived from a method that is deemed more accurate [10]. The advent of biomarker analysis now also offers an objective measure of intake, which may overcome the bias associated with self-reported data [11]. Biomarkers of both nutrient and food intake can be analyzed in plasma, serum, and urine to indicate both short- and long-term intake and can provide an objective validation of dietary assessment tools as they reflect, but are independent of food intake. Although a feasible validation tool, biomarker analysis is not always included in the validation of new dietary assessment techniques, which is perhaps in part due to the invasive nature of sample collection and associated cost. Another common validation reference used is direct observation of participants during eating occasions that can then be compared with reported or recalled dietary intake data [12,13]. However, this too can be costly and can often take place in a laboratory setting, potentially influencing an individual’s choices.

In a recent review of dietary assessment or tracker apps for mobile phones [14], the authors concluded that very few of the apps identified were based on scientifically valid nutrient composition databases and few had consulted nutrition professionals in the development process. With such unprecedented access to health and nutrition information the needs for scientifically validated, Web-based methods of dietary assessment are essential. The aim of this study was to describe the development, validation, and user evaluation of Ireland’s first Web-based, self-administered 24HDR tool “Foodbook24.”

## Methods

### Foodbook24

#### The Development of Foodbook24

The design of the Foodbook24 tool was informed by guidelines issued on the collection of dietary information that can be used to estimate nutrient intake and to assess exposure to biological agents and chemical substances by the European Food Safety Authority in 2009 [15]. In addition, interviews with key stakeholder organizations or institutions in Ireland and an extensive review of the literature concerning Web-based dietary assessment platforms were conducted to further inform the design of Foodbook24. The final proposed design of Foodbook24 was a self-administered, Web-based tool consisting of different independent components that facilitate the collection of dietary intake data without direct interaction with a researcher. These components include a screening and consent stage, demographic questionnaire, 2x24-hour multiple pass recall (administered on nonconsecutive days), food frequency, and food choice questionnaires, and finally a tool evaluation questionnaire. All of these stages occur at predetermined time points and have been developed independently of each other, meaning different parts of the tool could potentially be activated or deactivated depending on the requirements of any given survey or study.

**Figure 1:**
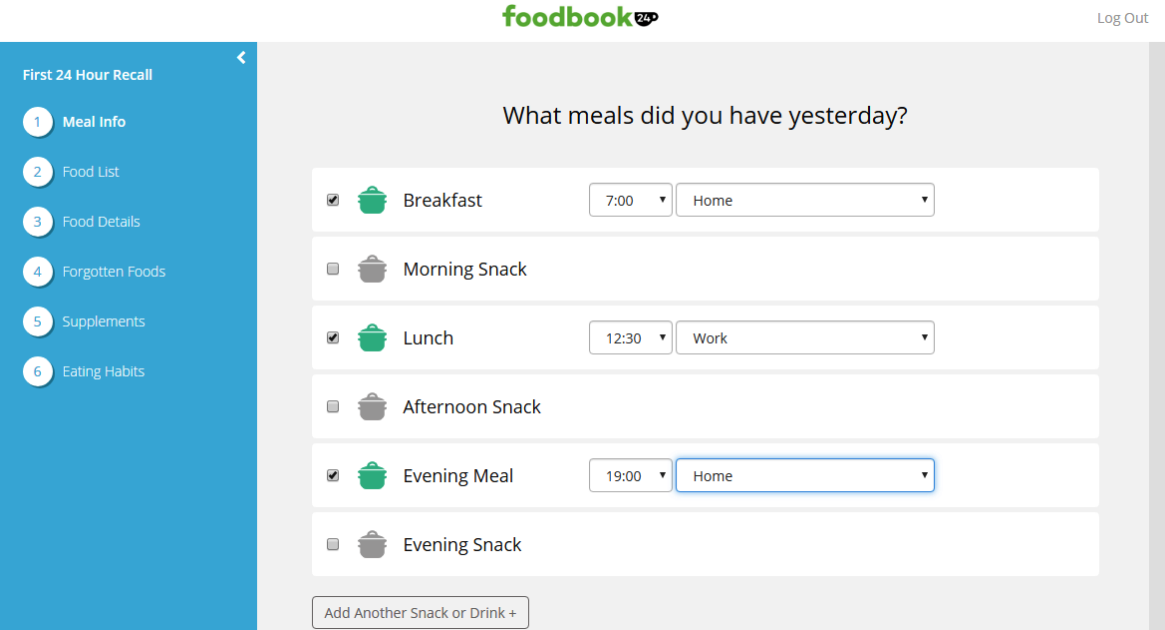
The meal information stage of Foodbook24.

For the dietary recall component of Foodbook24, the user is required to complete multiple passes (as described by Moshfegh et al [16]) to report their dietary intake for the previous 24-hour period. Initially, the user lists the meals and snacks consumed the previous day, reports the times that these meals were consumed (as depicted in Figure 1), and also the location of food preparation. The user then adds individual food and drink items to each of the defined meals or snacks using a free text search function to select food and drink items from a predefined database. Further questions known as “completeness of collection mechanisms” are presented to the user such as probe or linked food options and portion size information is then determined by selecting relevant amounts or portion size photographs. Finally, the user is presented with a review of selected items, a list of frequently forgotten foods, and queried about nutritional supplement intake and whether the reported intake was representative of usual intake. To populate the content of the Foodbook24 tool, various databases, completeness of collection mechanisms, and questionnaires were developed. These included a food list, nutrient composition, nutritional supplements and portion size databases, completeness of collection mechanisms for the 24-hour recall component and various supplementary questionnaires such as demographic and food choice questionnaires. The processes and considerations surrounding these components of the tool are described below.

##### Food List and Nutrient Composition

The food and drink list used in Foodbook24 is a shortened list of food and drinks consumed in the Irish National Adult Nutrition Survey (NANS 2008-2010) [17]. The food composition data linked to the NANS dataset are derived from UK food composition tables [18] and the Irish Food Composition Database (IFCDB) [19]. The reduction process of the list involved the merging of food codes of a similar description and/or composition [20]. The aim of the reduction process was to reduce the food list that participants would have to search through to describe their dietary intake, thus reducing participant burden without compromising the nutrient composition output. This process significantly reduced the total number of food and drinks from 2552 to 751 individual items. An investigation into the agreement of the shortened food list to the original comprehensive list is reported elsewhere [20], but overall shows excellent agreement and was therefore deemed appropriate for inclusion in Foodbook24. The food and drink items were grouped into 58 different food groups and further categorized into 18 categories.

##### Completeness of Collection Mechanisms

**Figure 2:**
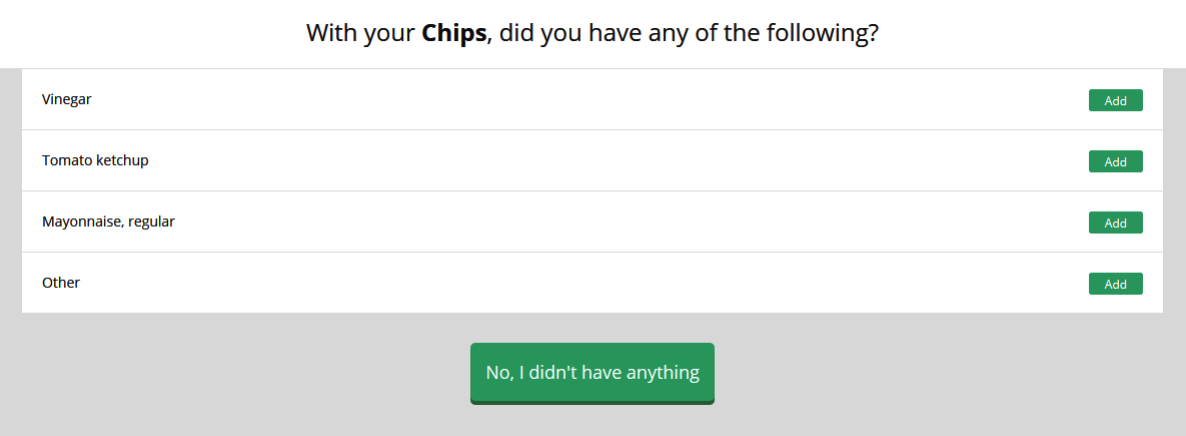
The “linked food” options available for the primary food selection of chips within Foodbook24.

**Figure 3:**
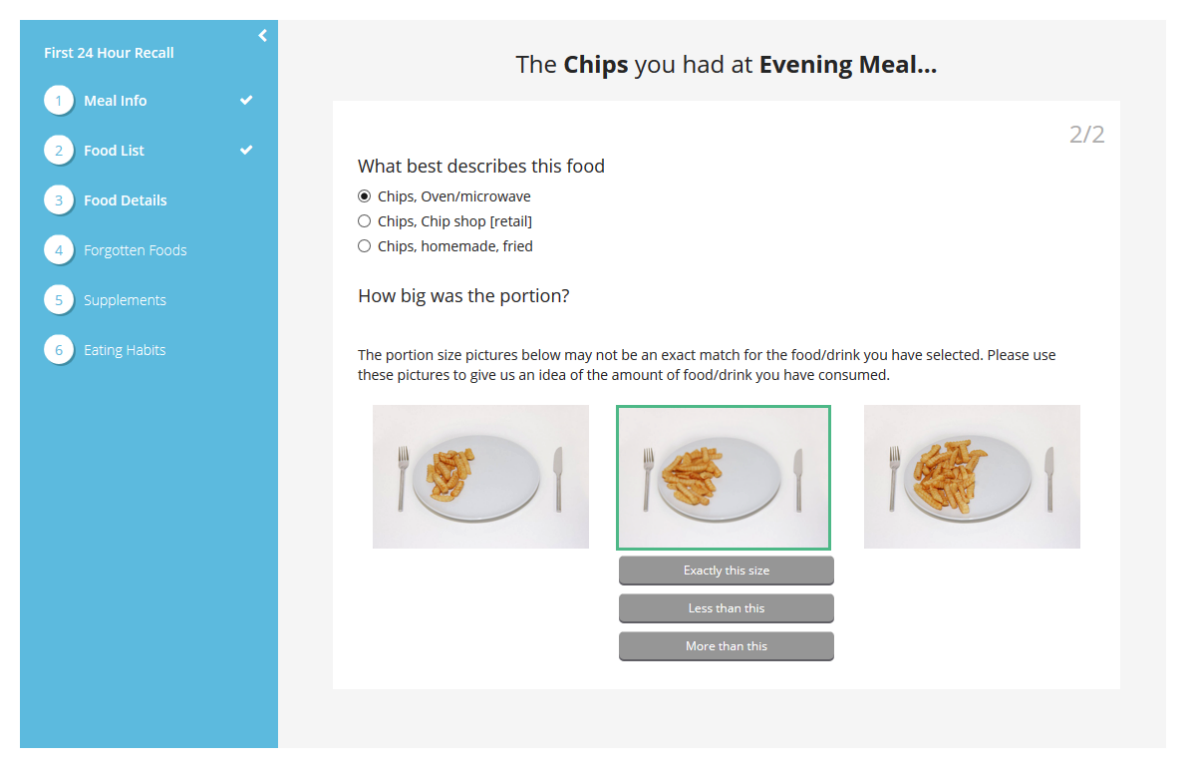
The food details stage of Foodbook24, presenting the user with a “probe question” and portion size images.

On review of Web-based 24HDR tools, the use of “probe” and “linked” (as described by Foster [21]) food options are commonly used to ensure the complete capture of dietary intake data. Linked food options were added to 132 food and drink items within the Foodbook24 food and drink list (an example of a linked food option is highlighted in Figure 2). These options are linked to the primary selection and are a list of options known to be commonly consumed with the primary selection. The use of “probe questions,” that is, questions posed to a respondent based on their primary food or drink selection provides more detail and further classifies that selection were implemented for 123 food and drink items (an example of a probe food question is depicted in Figure 3, where the user is asked to clarify whether the food item was homemade or retail). To improve the user experience of searching for food and drink items, “food tags” were applied to 484 of the 751 food and drink items. As the search function in this tool was based on the actual description of the food or drink item, “slang words” or brand names were tagged to the parent food to address common misspellings and multiple names of various food and drink items, for example, searching for “Houmus” would still retrieve “Hummus.”

##### Portion Size

Portion size can be estimated in two different ways within Foodbook24. Building on an existing dataset of portion size images (created as part of the Food4me project) [6], 96 additional portion size images were created for Foodbook24. The range of food and drink weights for which the portion size images depict was based on ranges of weights consumed in NANS [17]. For the majority of food and drink items, there are a set of 3 portion size images representing small, medium, and large portions (although these terms were not alluded to in the tool) as shown in Figure 3. The respondent also had the option to select “less than this” for any of the 3 images in a set, “exactly this size” or “greater than this.” Midpoint weights consumed in the NANS survey (eg, between small and medium portion size) underpin these options also. In Foodbook24, there are 174 sets of portion size images totaling 531 individual portion size images. For 195 food and drink items, average portion sizes from the Ministry for Agriculture, Fisheries and Food [22] were used. These are generally food items served in units such as cream crackers or biscuits; these were also followed by a question regarding how many serving of this item were consumed.

##### Nutritional Supplements

A database of 542 branded nutritional supplements with related nutrient composition was compiled to feature in the Foodbook24 tool. This nutritional supplement data consisted of supplements recorded as part of NANS [17] and those that were recorded as part of the Food4me study [6] by participants. These nutritional supplements were grouped into 26 supplement categories, for example, zinc supplements contains several different brands of zinc supplements for participants to choose from. An unknown or generic supplement composition option was also created using the median nutrient composition of all those supplements in that category.

##### Supplementary Questionnaires

The following components feature in the overall tool design; however, these components were not utilized in the validation study. A food frequency questionnaire (FFQ) was compiled for inclusion in the Foodbook24 tool based on EU Menu guidelines [15] and the Food4me FFQ [6]. The final questionnaire included 81 items and used the same frequency responses as featured in EPIC FFQ [23]; however, the Foodbook24 FFQ did not collect information on portion size. The FFQ was included in the tool to capture the intake of food and drink items less commonly consumed rather than contribute to nutrient intake. The food choice questionnaire [24] was also included as a multidimensional measure of motives related to food choice. The user is presented with a statement related to food choice such as, “It is important to me that the food I eat on a typical day contains a lot of vitamins and minerals” and can then agree or disagree with this using a 7-point scale. Screening, demographic, and evaluation questionnaires were built into the tool alongside a study information sheet and consent form, the contents of which can be easily changed depending on the use of the tool.

### Validation Study

#### Recruitment and Inclusion and Exclusion Criteria

Ethical approval for the study was obtained through University College Dublin (UCD) Human Research Ethics Committee (LS-15-27-Gibney-Timon). Participants were recruited via email using UCD mailing lists, University societies, and posters around campus. Individuals who expressed an interest in the study were contacted by phone and screened for eligibility. Subjects were eligible if the individuals were aged 18-64 years, fluent in English, had regular access to the Internet, were not pregnant, did not have any disease or condition that required chronic therapeutic nutritional or medical treatment, and had not been enrolled in or completed a degree, MSc or PhD in Human Nutrition. In total, 55 participants signed-up to take part in the study; however, 15 dropped out, which left a final sample size of 40.

#### Study Design

Participants were required to visit the Institute of Food and Health, UCD on three separate study visits during the study duration. At the first visit, informed consent, demographic information, and anthropometric measurements (including weight and body fat percentage and height) were collected. After the first visit, participants completed 3 nonconsecutive, unannounced, self-administered 24HDRs using the Web-based Foodbook24 tool. For this study, only the 24-h dietary component of the tool was used to record dietary intakes. Portion size photographs embedded in the tool depict a range of weights reported in NANS were used as portion size assessment aids. Emails were sent to participants on the morning and they were required to complete a recall using Foodbook24 without prior notice. In the middle of data collection using Foodbook24, participants attended study visit 2 and provided a fasting blood sample and a 24-hour urine collection. Following a 10-day wash out period, participants completed a 4-day semi-weighed food diary using a Tanita digital scale (KD-400) to weigh food and drink consumption as often as possible. On completion of this, participants attended the final study visit where they provided an additional fasting blood sample and 24-hour urine collection, completeness of check on their food diary, and completed a study evaluation questionnaire.

#### Collection of Biological Samples

Both blood and urine samples were collected from each participant in order to analyze specific biomarkers of nutrient intake. Blood samples (2x6 mL) were collected into lithium heparin tubes following a 12 h fasting period. Samples were spun for 15 min at relative centrifugal force (RCF) 1500 at 4°C. Plasma was transferred to labeled microtubes in 500 μL aliquots, two of which contained 10% meta-phosphoric acid (MPA) for the stabilization of ascorbic acid. All plasma samples were then frozen at −80°C. Plasma samples were analyzed by Vitas Analytical Services (Norway) for the determination of plasma ascorbic acid, carotenoids, and fatty acid content. Participants also provided a 24-hour urine collection and were instructed to collect the sample according to the protocol outlined in the National Diet and Nutrition Survey [25]. The urine samples were subsequently analyzed for urinary urea (as an indicator of protein intake [26]) using Daytona RX Clinical Analyzer (Randox, Nishinomiya, Japan) and urinary sodium, potassium, and creatinine were measured using the *Cobas Integra 700 Analyzer* (Roche Diagnostics) by the Department of Clinical Chemistry at St Vincent’s University Hospital. The ratio of observed over expected urinary creatinine excretion (UCE; creatinine index) [27] alongside other criteria such as reported >1 missed void and samples with a total volume <0.5 L was used to exclude incomplete 24-hour urine collections from biomarker analysis [28].

### Data and Statistical Analysis

Foodbook24 automatically generates a food and nutrient intake output for each user. The data from the semi-weighed food diaries was manually entered into WISP version 3 (Tinuviel Software, Anglesey, UK) by a single researcher in an attempt to maintain consistency and were reviewed independently by another researcher. Nutrient outputs for the semi-weighed food diaries were then generated. Mean daily nutrient intakes, standard deviations, and descriptive statistics (demographic data and evaluation questionnaire data) were computed in SPSS (version20) to determine the validity and user acceptability of Foodbook24. The normality of the data was assessed using the Shapiro-Wilk test before investigating the agreement between the dietary assessment methods, and parametric or nonparametric tests were used accordingly for subsequent analysis. Pearson and Spearman coefficient analyses was used to investigate the agreement between both methods in the reporting of nutrient intake, and to investigate the relationship between reported nutrient and food group intake and biological markers of intake. Correlation analysis was performed on energy adjusted data (nutrient intakes were energy-adjusted, that is, the percentage of energy intake for macronutrients and gram per milligram per milligram (g/mg/mg) per 10 MJ energy intake for micronutrients). Deattenuated correlation coefficients were also computed by multiplying the initial coefficient by *R*_1_, this was calculated as follows: R_1_=R_0_√(1 ((*sw*^2^) ⁄ (*sb*^2^)) ⁄ *n*), where (*sw*^2^) ⁄ (*sb*^2^) is the ratio of the within- and between-person variances and *n* is the number of replicates per person for the given variable. The within- and between-person variances were obtained from an analysis of variance (ANOVA) model. Correlations coefficients were considered as follows: very good (0.7 and greater), good (0.5-0.69), acceptable (0.3-0.49), and poor (0.3 or less) [29].

The relative agreement between Foodbook24 and the food diary was assessed using cross-classification of nutrient and food group intakes to estimate the percentage of participants who were classified by the two methods into quartiles of “exact agreement,” “exact agreement plus adjacent,” “disagreement,” and “extreme disagreement.” Bland and Altman [30] analysis was performed to assess the limits of agreement in the reporting of macronutrient intake, considering the two methods of dietary assessment to be comparable if greater than 95% of the data plots were within the limits of agreement. Wilcoxon Signed Rank and Paired Student *t* test were used to identify the differences in the nutrient intake, and independent samples *t* test were used to compare daily food group intakes reported by both methods.

### Evaluation of Foodbook24

Participants involved in two studies that investigated both the comparability of Foodbook24 (relative to an interviewer led 24-hour recall, results of which are not included in this publication) and the validity of Foodbook24 (compared with a 4-day semi-weighed food diary) were asked to complete an evaluation questionnaire once the study had concluded. In total, 118 participants (58 male and 60 female aged between 18 and 62 years) completed the optional questionnaire, 40 participants from the validation study and 79 from the comparison study. The design of the evaluation questionnaire was based around questionnaires used in similar studies that investigated the user acceptability of technology-based dietary assessment tools [31,32]. The questionnaire consisted of a 16-item evaluation questionnaire that was administered online. The focus of the questionnaire was to assess the participant’s overall experience using the 24-hour recall component of the tool only (as participants did not use other components of the tool, eg, FFQ as part of these studies), and their acceptability of some of the software design features, method preference, and future use.

## Results

### Study Population

A total of 55 participants signed-up to complete the validation study, of which 15 participants withdrew and therefore did not complete the entire study (dropout rate of 27%), with N=40 completing. Of those that withdrew, 9 reported that the collection of biological samples was too burdensome, 3 could not attend the study visits due to prior commitments, and 3 did not disclose their reason for dropping out. This left a final sample size of 40 participants that completed the study; however, 1 participant was excluded from the analysis as he or she did not follow the study protocol correctly. Table 1 displays the demographic characteristics of participants (n=39). The mean of age of participants was 32 years (age range 18-62 years). Over half of participants were either employed as staff (46.2%) or enrolled as students (10.3%) in UCD. The remainder of participants were either employed locally to the university or heard about the study through an Irish volunteer website.

**Table 1:** Demographic characteristics of participants.

Demographic characteristics		Mean (SD) or n (%)
**Age and BMI^a^, mean (SD)**			
	Age (years)	32.2 (13.4)
	BMI^a^ (kg/m²)	24.40 (3.75)
**Gender, n (%)**		
	Female	20 (51)
	Male	19 (49)
**Occupation, n (%)**		
	Student	4 (10)
	University staff	18 (46)
	Employed outside of the University	16 (41)
	Unemployed	1 (3)
**Smoking habits, n (%)**		
	Smoker	4 (10)
	Nonsmoker	29 (74)
	Ex-smoker	6 (16)
**Medical conditions, n (%)**		
	None	25 (65)
	One or more	14 (35)

^a^BMI: body mass index.

^b^SD: standard deviation.

#### Comparison of Nutrient Intake Reported by Both Methods of Dietary Assessment

The unadjusted, mean daily intakes for energy, nutrients, and food groups recorded using Foodbook24 and a semi-weighed food diary are displayed in Multimedia Appendix 1 and Table 2. The energy adjusted correlations, deattenuated correlations, mean difference, and the limits of agreement (2 standard deviations of the mean) between the two methods for the reporting of nutrients are also displayed in Multimedia Appendix 1. For nutrient intake, the majority of correlations between the dietary assessment methods ranged from acceptable to very good, and are statistically significant (range *r*=.32 to .75).

**Table 2:** Food group intakes recorded by participants using the Foodbook24 tool and a 4-day semi-weighed food diary.

Food group	Foodbook24 (g), mean (SD^a^)	Food diary (g), mean (SD)	*P* value
Grains, rice, pasta, and savories	207 (111.5)	171 (127.1)	.20
Bread and rolls	90.4 (54.54)	102 (46.29)	.31
Breakfast cereals	119 (83.9)	86.7 (80.99)	.09
Biscuits, cakes, and pastries	52.9 (36.67)	50.7 (44.68)	.82
Milk and yogurt	192 (228.4)	346 (223.4)	.05
Creams, ice creams, and desserts	67.0 (70.5)	73.7 (70.86)	.8
Cheeses	25.2 (13.40)	35 (16.1)	<.01^b^
Butter, spreading fats, and oils	14.93 (7.74)	10.42 (9.77)	<.05.^b^
Eggs and egg dishes	98.3 (69.61)	80.2 (52.68)	.26
Potatoes and potato dishes	135 (95.4)	147 (128.5)	.66
Veg and veg dishes	172 (107.5)	237 (152.5)	<.05^b^
Fruit and fruit dishes	372 (264.3)	252 (130.5)	<.01^b^
Fish and fish dishes	54.6 (58.23)	101 (102.0)	.05
Meat and meat products	244 (141.9)	249 (179.7)	.62
Alcoholic beverages	1314 (1208.2)	667 (761.5)	.09
Beverages other (sugar-sweetened)	1855 (1160.1)	1516 (854.7)	.14
Sugars, confectionary, preserves, and savory snacks	81.9 (50.81)	54.4 (47.63)	<.01^b^
Soups, sauces, and miscellaneous foods	91.8 (96.85)	130 (120.0)	.13
Nuts, seeds, herbs, and spices	16.8 (20.24)	37.4 (36.61)	<.001^b^

^a^SD: standard deviation.

^b^Significant difference in the reporting of food group intake between the two dietary assessment methodologies as defined by independent samples *t* test.

However, some correlation coefficients were not statistically significant including monounsaturated fat (*r*=.308, n=39, *P*=.05). Of the 34 nutrients investigated, there were significant differences between the reported mean intakes of 11 nutrients reported by the two methods; however, with the exception of protein (*P*=.02), there were no significant differences in the reporting of energy and macronutrient intake. Deattenuated correlation coefficients were higher, but the improvement was modest with the exception of intakes of fat (g/d).

**Figure 4:**
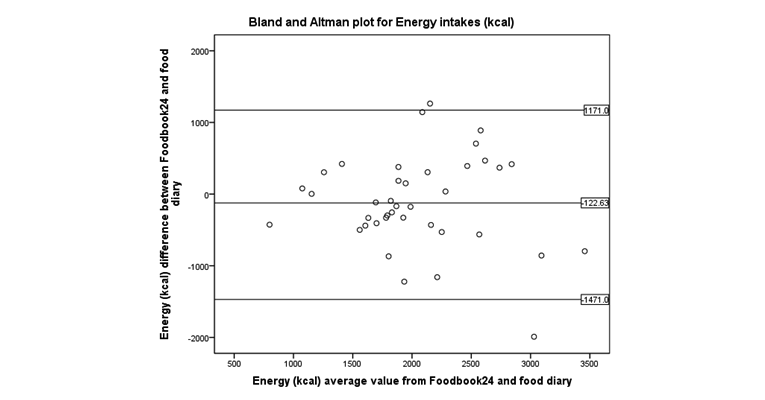
Bland and Altman plot examining the mean difference in reporting of energy intake by the two methods.

**Figure 5:**
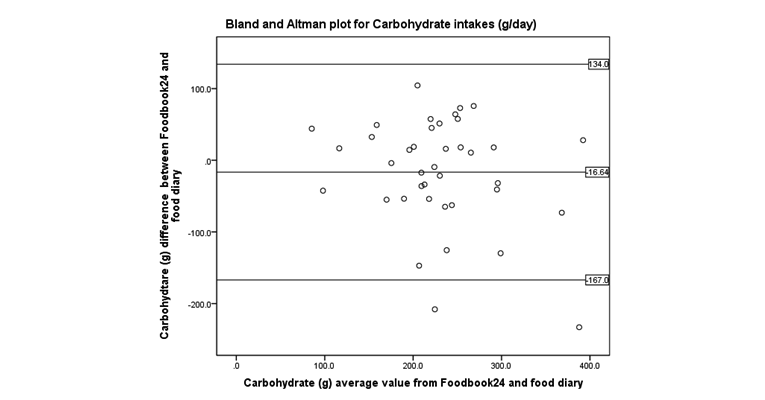
Bland and Altman plot examining the mean difference in reporting of carbohydrate intake by the two methods.

**Figure 6:**
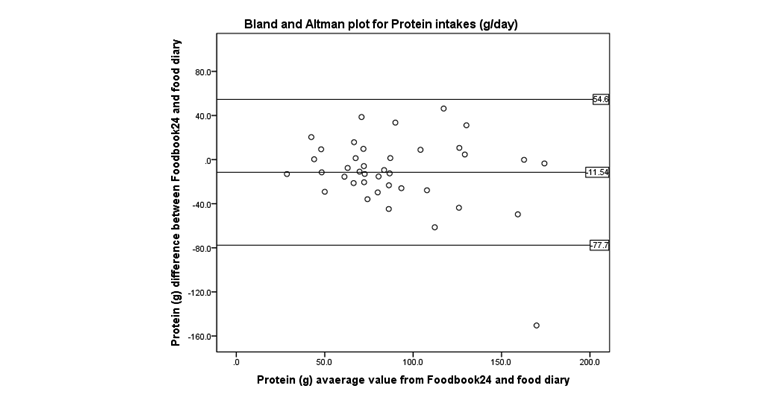
Bland and Altman plot examining the mean difference in reporting of protein intake by the two methods.

**Figure 7:**
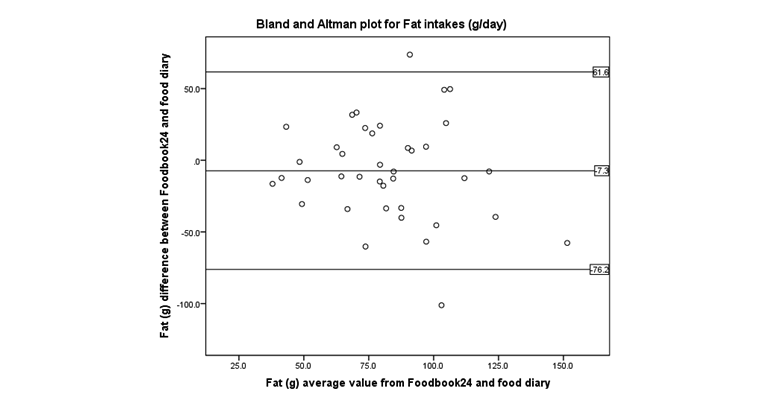
Bland and Altman plot examining the mean difference in reporting of fat intake by the two methods.

**Figure 8:**
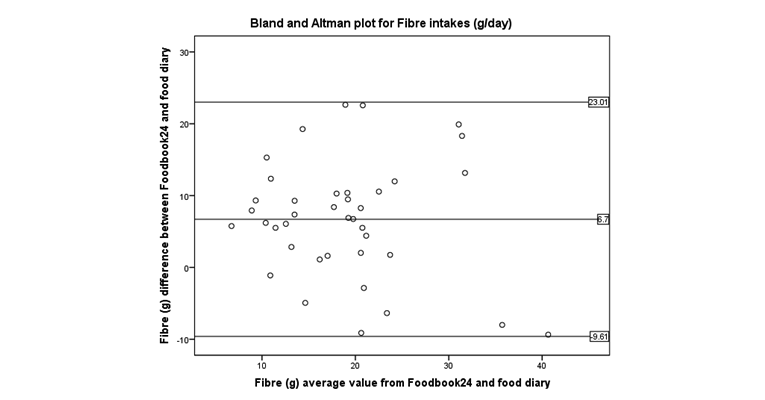
Bland and Altman plot examining the mean difference in reporting of fiber intake by the two methods.

Bland and Altman (Figures 4-8) analysis was used to further investigate the agreement between and the semi-weighed food diary. For macronutrients, Foodbook24 reported slightly lower intakes than the food diary; however, 95% or more of the data cases fell within the limits of agreement suggesting that the methods provide comparable intakes of these nutrients. The cross-classification of mean energy and nutrient intakes reported by the two methods are displayed in Table 3. The percentage of participants classified in “exact agreement” category of intake by both methods varied from 26% (% energy from saturated fat) to 74% (zinc). The majority of participants were classified in the “exact agreement and adjacent” category of intake by both methods with percentages varying from 69% (carotene) to 92% (zinc, potassium, and sodium). The percentages of participants that were classified into the “extreme disagreement” were low; and for some nutrients (protein, niacin, potassium, and sodium), no participants were classified into this category.

**Table 3:** Cross-classification of quartiles of mean energy and nutrient intake derived from Foodbook24 and a 4-day semi-weighed food diary.

Nutrient	Exact agreement (%)^a^	Exact agreement and adjacent (%)^b^	Disagreement (%)^c^	Extreme disagreement (%)^d^
Energy (kcal/d)	46.2	84.6	7.7	7.7
% Energy carbohydrate	30.8	76.9	20.5	2.6
% Energy protein	41.0	84.6	12.8	2.6
% Energy total fat	28.2	82.1	10.3	7.7
% Energy saturated fat	26.3	73.7	21.1	5.3
Protein (g/d)	56.4	84.6	7.7	0
Carbohydrate (g/d)	46.2	82.1	10.3	7.7
Sugars (g/d)	33.3	79.5	12.8	7.7
Starch (g/d)	43.6	84.6	10.3	5.1
Dietary fiber (g/d)	41.0	79.5	17.9	2.6
Fat (g/d)	38.5	74.4	17.9	7.7
Saturated fat (g/d)	43.6	76.9	17.9	5.1
Monounsaturated fat (g/d)	33.3	74.4	20.5	5.1
Polyunsaturated fat (g/d)	43.6	79.5	15.4	5.1
Retinol (µg/d)	30.8	71.8	20.5	7.7
Carotene (µg/d)	35.0	69.2	28.2	2.6
Vitamin D (µg/d)	33.3	87.2	10.3	2.6
Vitamin E (mg/d)	38.5	82.1	15.4	2.6
Riboflavin (mg/d)	43.6	83.7	12.8	2.6
Niacin (mg/d)	51.3	87.2	12.8	0
Vitamin B6 (mg/d)	41.0	84.6	12.8	2.6
Vitamin B12 (µg/d)	28.2	82.1	10.3	7.7
Folate (µg/d)	46.2	84.6	12.8	2.6
Vitamin C (mg/d)	41.0	79.5	12.8	7.7
Calcium (mg/d)	35.9	79.5	15.4	5.1
Magnesium (mg/d)	41.0	84.6	12.8	2.6
Iron (mg/d)	38.5	79.5	17.9	2.6
Copper (mg/d)	38.5	82.1	15.4	2.6
Zinc (mg/d)	74.4	92.3	5.1	2.6
Potassium (mg/d)	51.3	92.3	7.7	0
Sodium (mg/d)	46.2	92.3	7.7	0

^a^Exact agreement: percentage of cases cross-classified into the same quartile.

^b^Exact agreement and adjacent: percentage of cases cross-classified into the same or adjacent quartile.

^c^Disagreement: percentage of cases cross-classified 2 quartiles apart.

^d^Extreme disagreement: percentage of cases cross-classified 3 quartiles apart.

#### Comparison of Food Group Intakes Reported by Both Methods of Dietary Assessment

The mean food group intakes reported by both methods and the significant difference in the reporting of food group intake between the methods is presented in Table 2. Of the 19 food groups investigated, there were significant differences between the reporting of 6 food groups including “fruit and fruit dishes,” “alcoholic beverages,” and “sugars and confectionary, preserves, and savory snacks.” The cross-classification of mean food group intakes reported by the two methods is displayed in Table 4. The percentage of participants classified in “exact agreement” category of intake by both methods varied from 25.6% (“sugars, confectionary, preserves, and savory snacks” and “soups, sauces, and miscellaneous foods”) to 79% (“creams, ice creams, and desserts”). The majority of participants were classified in the “exact agreement and adjacent” category of intake by both methods; however, in the “creams, ice creams, and desserts” food group, no participants were classified into this category. Similar to the results for nutrients intakes, the percentages of participants that were classified into the “extreme disagreement” were low; and for some food groups (eg, alcoholic beverages), no participants were classified into this category.

#### Comparison of Biological Markers of Intake Against Food and Nutrient Intake Reported by Methods of Dietary Assessment

The relationships between urinary (recovery) and plasma (concentration) biomarkers and nutrient and food group intake recorded by both methods are reported in Table 5. Despite the researcher’s best efforts to recruit individuals that did not take supplements, this was not possible in every case. The researchers did ask participants not to take nutritional supplements during the study, where possible. As a result, participants who reported taking supplements, including protein, multivitamin, vitamin, mineral and/or fish oil, before the provision of blood and urine samples during this study were excluded from analysis. This excluded 15 participants from plasma biomarker analysis (resulting in n=24 for plasma biomarker analysis) and 11 participants from urinary biomarker analysis (resulting in n=28 for urinary biomarker analysis). With the exception of comparison of fruit and vegetable intakes (g/d) derived from Foodbook24 and total plasma carotenoids (*r*=.315, n=28, *P*=.10), there were good, significant correlations (*r*=.42 to .64) between food group and nutrient intakes reported by Foodbook24 and biomarkers of nutrient and food group intake from plasma and urine samples. Nutrient and food group intakes derived from the semi-weighed food diary compared with biomarkers from urine and plasma samples resulted in strong, significant correlations except in the case of urinary potassium.

**Table 4:** Cross-classification of quartiles of mean food group intake derived from Foodbook24 and a 4-day semi-weighed food diary.

Food group	Exact agreement (%)^a^	Exact agreement and adjacent (%)^b^	Disagreement (%)^c^	Extreme disagreement (%)^d^
Grains, rice, pasta, and savories	43.6	71.8	17.9	10.3
Bread and rolls	35.9	82.1	7.7	10.3
Breakfast cereals	28.2	71.8	17.9	10.3
Biscuits, cakes, and pastries	28.2	61.5	30.8	5.1
Milk and yogurt	35.9	79.5	17.9	2.6
Creams, ice creams, and desserts	79.5	0	20.5	0
Cheeses	48.7	74.4	12.8	12.8
Butter, spreading fats, and oils	38.5	66.7	25.6	7.7
Eggs and egg dishes	48.7	71.8	25.6	2.6
Potatoes and potato dishes	38.5	76.9	20.5	2.6
Veg and veg dishes	46.2	87.2	10.3	2.6
Fruit and fruit dishes	35.9	79.5	17.9	2.6
Fish and fish dishes	51.3	79.5	20.5	0
Meat and meat products	61.5	92.3	7.7	0
Alcoholic beverages	63.2	15.8	21.0	0
Beverages other (sugar-sweetened)	48.7	41.0	10.3	0
Sugars, confectionary, preserves, and savory snacks	25.6	71.8	17.9	10.3
Soups, sauces, and miscellaneous foods	25.6	76.9	12.8	10.3
Nuts, seeds, herbs, and spices	35.9	74.4	17.9	7.7

^a^Exact agreement: percentage of cases cross-classified into the same quartile.

^b^Exact agreement and adjacent: percentage of cases cross-classified into the same or adjacent quartile.

^c^Disagreement: percentage of cases cross-classified 2 quartiles apart.

^d^Extreme disagreement: percentage of cases cross-classified 3 quartiles apart.

**Table 5:** Biomarker and food group relationship (as derived from Foodbook24 and food diary).

Biomarkers^a^		Nutrient or food group^b^	Foodbook24 correlation coefficient	Diary correlation coefficient
			(*r* value)^c^	(*r* value)^c^
**Concentration biomarkers**			(n=28)	(n=34)
	Plasma ascorbic acid (µM)	Fruit and veg (g/d)	0.421	0.505
	Plasma total carotenoids (µmol/L)	Fruit and veg (g/d)	0.315^e^	0.671
	Plasma omega-3 index	Fish (g/d)	0.468	0.769
	Plasma ascorbic acid (µM)	Vitamin C (mg/d)	0.518	0.605
**Recovery biomarkers**			(n=34)	(n=33)
	Urinary urea (mmol/d)	Protein (g/d)	0.645	0.824
	Urinary potassium (mmol/d)	Potassium (mg/d)	0.542	0.269^e^
	Urinary sodium (mmol/d)^d^	Sodium (mg/d)	0.56	0.476

^a^Plasma and urinary biomarkers identified from fasted blood samples and 24-hour urine collections (respectively) collected from participants directly after recording intake using either dietary assessment methods.

^b^The data refer to nutrient and food group intakes recorded using Foodbook24 and the semi-weighed food diary.

^c^Spearman correlation coefficient.

^d^Sodium excretion measures were corrected to account for 90% excretion of all sodium consumed (28).

^e^Not statistically significant correlations (*P*<.05).

### User Evaluation of Foodbook24

The main results of participants’ evaluation of the 24-hour recall component of Foodbook24 are depicted in Table 6. The majority of respondents were very positive in their evaluation of Foodbook24. Overall, the majority found the Foodbook24 system user-friendly with 69.5% (82/118) reporting it easy or “OK” to use. When asked if participants felt that Foodbook24 changed what they ate and drank, a majority of 62.7% (74/118) felt it did not change at all, whereas 34.7% (41/118) felt it changed it a little, and 2.5% (3/118) felt it changed a lot. Importantly, when asked if there were any foods or drinks that participants did not want to record, a majority of 95.8% (113/118) stated “no.”

**Table 6:** Participant acceptability of Foodbook24.

Question posed to participant	Participant responses			
**Impact of Foodbook24 on diet**	Changed a lot (%)	Changed a little (%)	Did not change at all (%)	
	2.5	34.7	62.7	
**Completion time**	Too long (%)	Okay (%)	Short (%)	Very short (%)
	6.8	63.6	22.9	6.8
**User friendliness**	Difficult (%)	Okay (%)	Easy (%)	Very easy (%)
	3.4	33.1	36.4	27.4
**Remembering to use Foodbook24**	Difficult (%)	Okay (%)	Easy (%)	Very easy (%)
	6.8	38.1	40.7	14.4
**Preferred method**	Foodbook24 (%)	Reference method^a^ (%)	Other (%)	
	67.8	31.4	0.8	
**Use Foodbook24 for longer**	1 week (%)	1 month (%)	6 months (%)	No (%)
	27.1	30.5	24.6	17.8

^a^Two different reference methods for comparison and validation study. This equates to 38.5% (30/78) in favor of the traditional interview-led 24HDR and 17.5% (7/40) for semi-weighed food diary.

Participants were asked about using Foodbook24 for longer periods of time to gain insight into the potential long-term use of the tool. The results were favorable for the shorter time of a week (considering the completion of two 24-hour recalls per week) with 82.2% willing to use Foodbook24 for a week, persistence understandably decreased with 55.1% willing to use for a month, and 24.6% for 6 months. When asked to select which method participants would prefer to use in future (Foodbook24 vs the respective reference method), 67.8% (80/118) opted for Foodbook24 and 31.4% (27/118) for the reference method. The researchers were aware that these particular results may have been influenced by the different reference method used in either study (interviewer-led 24HDR in the comparison study and a 4-day semi-weighed food diary in the validation study). The participant burden associated with a 4-day semi-weighed food diary may have been greater than that of an interview-led 24HDR. As a result, responses were split by study involvement. A majority of 61.5% (48/78) opted for Foodbook24 when compared with 38.5% (30/78) in favor of the interview-led 24HDR. Foodbook24 was an even clearer preference when compared with the semi-weighed food diary with 80% (32/40) in favor of Foodbook24 as opposed to 17.5% (7/40) for semi-weighed food diary and 1 participant (2.5%) opted for “other.”

## Discussion

### Principal Findings

As far as the researchers are aware, Foodbook24 is the first Web-based dietary assessment tool developed to estimate food and nutrient intakes specifically for Irish adults. The results of this investigation into the validity of Foodbook24 suggest that the tool provides nutrient and food group intake estimates comparable with that of a semi-weighed food diary. The use of an objective measure of validity; biological markers of nutrient intake in blood and urine samples further confirm this agreement between methods.

With regards to food group intake, the results of the paired *t* test and cross-classification analysis indicate that there is good agreement between the two methods in the reporting of the majority of food group intakes. Interestingly Foodbook24 reported higher intakes of food groups that are perceived unhealthy such as “alcoholic beverages” and “sugars and confectionary” compared with the food diary. This may be due to variation in diet as was the case for fiber intakes. However, the lack of face-to-face interaction between participant and researcher that is encountered on the completion of a food record (food record review with researcher) may encourage participants of Foodbook24 to report their intake with less inhibition or in a less inhibited manner [33,34]. This may highlight an advantage of Web-based dietary assessment in terms of attenuating the influence of social desirability when self-reporting dietary intake. A study conducted by Probst and Tapsell [35] found that patients using a computer to self-report intake were more willing to report all foods eaten to the computer than to a dietitian. Intakes of “milk and yogurt” were lower with Foodbook24 compared with the food diary, although these differences were not statistically significant. Although milk was a linked food (to prompt the participant to record milk with items such as cereal and hot beverages), it may be the case that milk consumed as a beverage was frequently forgotten when recording dietary intake using Foodbook24.

Overall for nutrient intakes, correlations were acceptable to very good; however, there were few significant differences between nutrient intakes reported using Foodbook24 and the semi-weighed food diary. The correlation ranges observed were also comparable with other studies investigating the validity of Web-based 24-hour recall methods [36-39]. There were, however, nutrients that were not correlated such as the intakes of monounsaturated fat and intakes that were significantly different from the semi-weighed food diary, for example, protein and fiber. In an evaluation of the shortened food-list (n=751) for integration into Foodbook24 [20], it was observed that there was less agreement for mean daily intake of monounsaturated and saturated fat due to the changes in food composition data that resulted from merging similar food and drink items that had similar composition into single food or drink descriptors or codes. Expanding the number of food items within this category may improve the agreement between methods for these nutrients.

Foodbook24 reported lower intakes of protein compared with the semi-weighed food diary, potentially due to different portion size estimations (portion size photographs using Foodbook24 compared with free weight entry using semi-weighed food diary). The within-person variance (ie, day-to-day variation in diet) during the two different data collection time points also accounted for the differences of intakes reported by both methods for fiber. Food items with high fiber content, for example, baked beans in tomato sauce, fruit smoothies, and breakfast cereals were more frequently reported by participants recording dietary intake using Foodbook24 than with the food diary. The challenges of the variation of diet during dietary assessment validation whereby two separate methods (the test and references measure) assess dietary intakes over two different time points have been noted by others [40,41]. However, despite the differences in fiber intake recorded, none of the cases fell outside of the limits of agreement in the Bland and Altman plot for fiber intake (Figure 8) suggesting that there may be an acceptable level of agreement between the two methods.

Biological markers of nutrient intake can serve as an objective validation of dietary assessment methods as they reflect nutritional status, metabolism, and recent dietary intake, but the error associated with biological markers is independent of dietary intake assessment error [42]. Urinary urea excretion was used as an independent marker of protein intake in this study as it can be assumed that urinary urea is excreted in constant proportion to urinary nitrogen for individuals in energy balance and consuming a westernized diet [26]. Overall, a slightly stronger correlation was observed for intakes derived from the food diary compared with Foodbook24, but this was to be expected considering more accurate portion size assessment observed with semi-weighed food records [43]. Dietary intakes recorded by both methods correlated significantly with recovery biomarkers (urinary urea, potassium, and sodium) with the exception of urinary potassium, which did not significantly correlate with potassium intakes reported from the food diary but did with dietary intakes reported from Foodbook24. This was an unexpected, but promising finding considering potassium is present in a large variety of food groups and is considered a reliable recovery biomarker in dietary studies [44]. Concentration biomarkers can be used to assess which assessment method yielded the most reliable estimates of intakes [45]. The food diary provided more reliable intakes of fish intake and total carotenoids, but estimates were similar for fruit and vegetable intake and ascorbic acid suggesting that both methods are valid and comparable in the reporting of these dietary components. The correlation coefficients between nutrient intakes and biomarkers of nutrient intakes reported in this study are comparable with those reported in other validation studies [46-48], although the correlations between protein intake and urinary urea observed in this study were stronger than those reported in the pooled results from 5 validation studies of dietary self-report instruments [49]. The pooled results study reported an average correlation coefficient for reported protein intakes versus true protein intakes of *r*=.29 when assessed using an FFQ and *r*=.48 when assessed using the average of three 24-hour recalls. However, urinary nitrogen was used as a biomarker of protein intake for these studies so that a direct comparison cannot be made. Overall, these results indicate that self-administered 24HDR via Foodbook24 provide estimates of certain nutrients and fruit and vegetable intakes similar to that of a 4-day semi-weighed food diary.

The majority of participants who used Foodbook24 were enthusiastic in their evaluation of the Web-based tool, and a large proportion of respondents claimed that they would be willing to use Foodbook24 for a week. Freese et al [50] reported similar positive responses where 95% of 370 adult participants would be willing to repeat the Web-based 24HDR after completing 3 recalls. In contrast, Maes et al [31] reported that adolescent participants involved in the HELENA (Healthy Lifestyle in Europe by Nutrition in Adolescence) “Food-O-Meter” project were not eager to use their computer-based FFQ more than once. Most importantly, Foodbook24 was the preferred method of dietary intake assessment for the majority of participants. Vereecken et al [51] reported a similar preference for an online method with 73% of parents in the Children's and Adolescents' Nutrition Assessment and Advice on the Web (CANAA-W) study stating that they preferred a 3-day computerized food record over the paper and pencil 3-day food record (12%), whereas 10% selected a computerized FFQ and 6% selected a paper and pencil FFQ. Similarly, Monnerie et al [52] reported a 77% (77/100) preference for online assessment versus a traditional diary over 7 days. Thompson et al [53] also observed a clear participant preference for ASA24 when compared with the traditional interviewer administered method across a range of age groups (20-70 years) and education levels. This may highlight a willingness among Irish adults to record their dietary intake with the aid of technology and as such offers hope that Web-based methods can act as a viable alternative or accompaniment to nutritional surveillance in Ireland. Future research is required to ascertain the actual potential for Web-based innovations to work in tandem with current methods in nutritional surveillance.

### Strengths and Limitations

Although this study has many strengths including the use of biomarkers of intake in the tool validation and the inclusion of nationally representative food intake data in the tool design, it is also important to consider the study limitations. The small sample size recruited was a limitation of this study and the exclusion of participants that took nutritional supplements further reduced numbers for certain aspects of analysis. Unfortunately, high dropout rates as observed with this study are commonly reported in studies that require participants to provide biological samples on more than one occasion, particularly 24-hour urine collections. With regards to the analysis of urine samples, the use of urinary nitrogen and para-aminobenzoic acid (*PABA*) would have been preferable as an objective measure of protein intake and as a check for completeness of collection, respectively; however, these measurements were not possible for this study. Finally, the majority of participants recruited as part of the validation study and in other studies evaluating Foodbook24 were young, healthy, and motivated individuals, and therefore may not represent the opinions of the general adult population with respects to their ability to use Foodbook24 and their preference of dietary assessment methods. To further evaluate Foodbook24, a proof-of-principle (PoP) study that involves Foodbook24 being made freely available to the general Irish adult population is currently underway. The PoP study will provide insight into the acceptability of Foodbook24 with a more representative sample of the general Irish adult population.

### Conclusions

In this paper, we investigated the relative validity of a Web-based 24HDR tool, Foodbook24. Although this study only investigates the validity of the 24HDR component of the Foodbook24 tool, the tool itself incorporates the use of blended assessment methods that has the potential to yield more accurate data on habitual intake [5]. This study describes the robust validation of Foodbook24 against a semi-weighed food diary and biological markers of nutrient and food group intake. The results from this study demonstrate that Foodbook24 performs well when compared with a semi-weighed food diary and provides comparable estimates of food and nutrient intakes. A major advantage of Foodbook24 and of similar Web-based dietary assessment tools is the reduced cost associated with the collection of dietary intake data compared with traditional methods. More importantly, Web-based methodologies facilitate the collection of data in a neutral environment, in the absence of a researcher with less burden for the participant which may encourage participants to report intake more honestly. Participant acceptability data gathered so far suggests Foodbook24 was well received by the majority of participants in this study sample which indicates the potential of Foodbook24 for use in nutrition related research or as a means of intermittent data collection between national nutrition surveys in Ireland.

## Appendix

Multimedia Appendix 1 Daily energy and nutrient intakes recorded by participants using the Foodbook24 tool and a 4-day semi-weighed food diary.

## Abbreviations

ANOVA: analysis of variance
FFQ: food frequency questionnaire
24HDR: 24-hour dietary recall
RCF: relative centrifugal force
UCE: urinary creatinine excretion
