# Supplementary material for: The Development, Validation, and User Evaluation of Foodbook24: A Web-Based Dietary Assessment Tool Developed for the Irish Adult Population
Source: J Med Internet Res. 2017 May 11;19(5):e158. doi: 10.2196/jmir.6407 (PMC5445234; doi:10.2196/jmir.6407)
Supplement: Multimedia Appendix 1 [file jmir_v19i5e158_app1.pdf]

Multimedia appendix 1: Daily energy and nutrient intakes recorded by participants using the Foodbook24 tool and a 4-day semi-weighed food diary.

Nutrient	Foodbook24 mean (SD[a])	Food diary mean (SD)	Correlation coefficient (*r* value)[b]	Deattenuated correlation coefficient (*r* deatt)[c]	Mean diff	*P* value	Limits of agreement[d]
**Energy (kcal/d)[e]**	1971 (626.6)	2100 (679.0)	.536	.565	−122	.168	−1417/1171
**% Energy carbohydrate[e]**	45.7 (8.09)	45.4 (8.05)	.364	.386	0.22	.806	−16.73/17.17
**% Energy protein[e]**	16.7 (3.83)	17.8 (5.09)	.478	.505	−1.16	.093	−10.19/7.87
**% Energy fat[e]**	36.1 (7.72)	36.6 (6.06)	.328	.467	−0.53	.669	−17.27/16.22
**% Energy saturated fat[e]**	14.3 (3.83)	12.2 (3.18)	.335	.369	2.04	.004[f]	−6.25/10.32
**Protein (g/d)[e]**	83.5 (33.27)	95.0 (44.02)	.750	.778	−11.54	<.05[f]	−77.7/54.6
**Carbohydrate (g/d)[g]**	221 (67.1)	238 (84.5)	.525	.553	−16.64	.177	−167/134
**Sugars (g/d)[g]**	96.9 (42.83)	104 (39.3)	.356	.383	−7.89	.299	−101/85.5
**Starch (g/d)[g]**	119 (42.2)	126 (54.2)	.606	.632	−6.95	.332	−95.2/81.3
**Dietary fiber (g/d)[e]**	22.2 (8.10)	15.5 (9.08)	.519	.559	6.70	<.001[f]	−9.61/23.01
**Fat (g/d)[g]**	78.4 (27.38)	85.7 (31.85)	.330	.577	−7.30	.194	−76.2/61.6
**Saturated fat (g/d)[e]**	31.1 (12.28)	28.7 (11.96)	.385	.413	2.38	.27	−24.5/29.2
**Monounsaturated fat (g/d)[e]**	28.4 (10.33)	29.6 (12.61)	.308[h]	.335	−1.22	.645	−28.7/26.3
**Polyunsaturated fat (g/d)[e]**	13.9 (5.63)	14.2 (5.88)	.448	.476	−0.30	.867	−12.51/11.91
**Carotene (µg/d)[e]**	4973 (3917.5)	6832 (7434.1)	.316	.320	−1859	.103	−13078/9360
**Vitamin D (µg/d)[e]**	2.7 (1.93)	3.0 (2.57)	.415	.453	−0.28	.586	−5.34/4.79
**Vitamin E (mg/d)[e]**	10.8 (4.53)	10.0 (4.95)	.624	.650	0.78	.097	−8.99/10.56
**Riboflavin (mg/d)[e]**	1.6 (0.75)	2.0 (0.87)	.336	.366	−0.36	.004	−1.97/1.26
**Niacin (mg/d)[e]**	23.4 (11.97)	25.9 (15.47)	.282[h]	.332	−2.57	.35	−28.7/23.6
**Vitamin B12 (µg/d)[e]**	4.4 (3.26)	4.7 (3.00)	.132	.149	−0.32	.418	−7.63/6.99
**Folate (µg/d)[e]**	277 (102.8)	302 (144.7)	.446	.463	−24.59	.097	−259/210
**Vitamin C (mg/d)[e]**	115 (74.9)	129 (79.8)	.272[h]	.288	−14.36	.418	−190/161
**Calcium (mg/d)[e]**	869 (360.8)	972 (390.5)	.435	.454	−102	.135	−808/603
**Iron (mg/d)[g]**	11.9 (4.09)	13.8 (5.24)	.440	.469	−1.90	<.05[f]	−11.36/7.57
**Potassium (mg/d)[e]**	3139 (1155.9)	3577 (1236.5)	.554	.582	−437	<.05 [f]	−2376/1500
**Sodium (mg/d)[e]**	2265 (875.0)	2552 (991.3)	.304[h]	.327	−286	<.05 [f]	−1840/1268

[a]SD: standard deviation.

[b]Correlations performed using energy adjusted values for macro and micronutrient intake.

[c]The corresponding deattenuated correlation coefficients were then calculated by multiplying the initial coefficient by R_1, was calculated as follows: $R_1=R_0\sqrt{(1 + ((sw^2)/(sb^2))/n)}$, where $(sw^2)/(sb^2)$ is the ratio

of the within- and between-person variances and n is the number of replicates per person for the given variable. The within- and between-person variances were obtained from an ANOVA model.

[d]Lower and upper limits of agreement (mean difference [2 SD]).

[e]Nonparametric data (Spearman correlation coefficient and Wilcoxon signed rank used).

[f]Significant difference in the reporting of a nutrient by the two dietary assessment methodologies.

[g]Parametric data (Pearson correlation coefficient and Paired t test used).

[h]Not statistically significant correlations. $P<.05$.
